# Prevalence of Self-Reported Stroke and Disability in the French Adult Population: A Transversal Study

**DOI:** 10.1371/journal.pone.0115375

**Published:** 2014-12-18

**Authors:** Alexis Schnitzler, France Woimant, Philippe Tuppin, Christine de Peretti

**Affiliations:** 1 Physical Medicine and Rehabilitation Department, Hôpital Raymond Poincaré - Assistance Publique – Hôpitaux de Paris, Université de Versailles Saint Quentin (EA 4497), Garches, France; 2 Agence régionale de santé d′Ile de France, Paris, France; 3 Caisse nationale d′assurance maladie des travailleurs salariés, Paris, France; 4 Institut de veille sanitaire, Saint-Maurice, France; 5 Hôpital Lariboisière - Assistance Publique – Hôpitaux de Paris, Paris, France; Fraunhofer Institute for Cell Therapy and Immunology, Germany

## Abstract

In France, the prevalence of stroke and the level of disability of stroke survivors are little known. The aim of this study was to evaluate functional limitations in adults at home and in institutions, with and without self-reported stroke. A survey named “the Disability Health survey” was carried out in people's homes (DHH) and in institutions (DHI). Medical history and functional level (activities-of-daily-living, ADL and instrumented-activities-of-daily-living IADL) were collected through interviews. The modified Rankin score (mRS) and the level of dependence and disability were compared between participants with and without stroke. 33896 subjects responded. The overall prevalence of stroke was 1.6% (CI95% [1.4%–1.7%]). The mRS was over 2 for 34.4% of participants with stroke (28.7% of participants at home and 87.8% of participants in institutions) versus respectively 3.9%, 3.1% and 71.6% without stroke. Difficulty washing was the most frequently reported ADL for those with stroke (30.6% versus 3% for those without stroke). Difficulty with ADL and IADL increased with age but the relative risk was higher below the age of 60 (17 to 25) than over 85 years (1.5 to 2.2), depending on the ADL. In the overall population, 22.6% of those confined to bed or chair reported a history of stroke. These results thus demonstrate a high national prevalence of stroke. Older people are highly dependent, irrespective of stroke history and the relative risk of dependence in young subjects with a history of stroke is high compared with those without.

## Introduction

Stroke is a common and serious pathology. It is the third cause of mortality in the world [1] and one of the major causes of disability. In France, the Dijon registry reported an incidence of first ever stroke of 113 per 100 000 inhabitants [2]. The number of patients admitted to hospital in 2008 for stroke (either first ever or further stroke) was near 100 000, equivalent to 152 per 100 000 inhabitants [3], [4].

The level of mortality following stroke in France is among the ten lowest in the world (51.1 per 100 000 inhabitants in 2009) [5], [6]. Most studies on the prevalence of stroke are regional studies and tend to be focused on the older population [7]–[9]. In the world, the prevalence of stroke is 62 million and is expected to reach 77 million in 2030 [7].

Across all age groups, the prevalence of stroke is estimated to be between 1.5% and 2.6% in industrialized countries [9]. In France, the first results of the ‘Disability Health at Home’ (DHH) and ‘Disability Health in Institutions’ (DHI) surveys showed a lower prevalence of people who reported a history of stroke than that described in other industrialized countries (1.2% across all age groups) [10].

Stroke survivors are frequently left with neurological sequelae. Two recent cohort studies in New Zealand and the United Kingdom found that 20–30% of patients were dependent following stroke, with a modified Rankin score (mRS) of 3 or higher (Moderate disability; requiring some help, but able to walk without assistance) 5–10 years following stroke [11], [12].

In France, the prevalence of stroke and particularly the level of disability in stroke survivors, are little known. The aim of this study was to evaluate and compare functional limitations in elementary and instrumental activities of daily living (ADL and IADL) in French subjects aged over 18 years, living at home and in institutions, with and without a history of stroke. The modified Rankin Scale (mRS) was used to compare levels of independence and to estimate the impact of a history of stroke on dependence in the whole of the French adult population.

## Method

### Disability Health surveys

The aim of the ‘Disability health’ survey system, coordinated by the Insee (Institut national de la statistique et des études économiques: National Institute of Statistics and Economic Studies) and the Drees (Direction de la recherche, des études, de l′évaluation et des statistiques: Directorate of research, studies, evaluation and statistics) is to estimate the number of dependent or disabled people in the general French population, to describe the functional health status of the population in general and to document the incidence of different types of disability (physical, mental, cognitive, psychological or multiple disabilities). The secondary aim is to describe the environmental factors which favor or limit activities of daily living based on the international classification of function (ICF). These detailed surveys thus explore social participation (work, family, social relations, leisure activities), discrimination and administrative recognition of disability and human, technical and financial assistance. The methodology used in the disability health survey has been described in detail elsewhere [13]. For the present study, data from two parts of the survey were analyzed: Disability Health at home, carried out in 2008, which covered the population living at home, and Disability Health in institutions which covered the population living in long-term institutions (2009). The study was on a national level.

### DHH survey

A preliminary survey was carried out prior to the Disability Health at Home survey. This survey was carried out in 2007 and was called ‘Daily Life and Health (DLH)’. A questionnaire was posted to 127 200 homes which were randomly drawn from the French census. The questionnaire consisted of 26 questions of which 13 were related to functional limitations, 1 to global activity limitations and 1 to the perception of disability. The response rate was 80% (102 000 homes, including 238 000 subjects). The survey base was then built from the responders of the preliminary survey. It consisted of four strata according to the severity of the reported level of disability (no disability, slight, moderate or severe). In order to over-represent disabled people, the frequency of the survey increased with the level of disability in the stratum. The survey was declarative with a face to face interview. The level of participation in the interviews was 77%.

### DHI survey

This complementary survey was carried out in institutions for elderly people (long term health care units, establishments for the dependent elderly and retirement homes), institutions for disabled adults as well as in psychiatric institutions and centers for social reintegration. Of the 1567 institutions sampled across the whole of France, 97% agreed to participate in the survey. The level of participation of institutionalized subjects was 90.9%.

### Questionnaire and constitution of the database

The interview questionnaire was practically identical for both surveys. Medical history was sought via the question “Do you have, or have you ever had one of these illnesses or health problems?” with a list of 51 diseases including stroke (“cerebro vascular accident, stroke, brain hemorrhage, brain thrombosis”). In the case of a positive result, the subject, or a relation or caregiver if the subject was unable to reply, was then questioned regarding any sequelae from the stroke. The questions regarding limitations of motor function and basic activities of daily living (ADL) included in all the surveys were used to compare the functional level of the subjects who reported a history of stroke with that of subjects who did not report a history of stroke. Four ADL (bathing, dressing, feeding, and continence) and 4 IADL (difficulty preparing a meal, carrying out administrative processes independently, managing medication and walking from one room to another) were evaluated.

### Ethics statement

This study was planned as a research project. It was performed in collaboration with the French National Institute of Statistics. This study was declared of public interest by the CNIS (Conseil National d′Information Statistique) and was approved by the CNIL (Commission Nationale de l′Informatique et des Liberte's, French law no. 78–17). According to the French law, written informed consent is not required for this type of study. The data used were taken from the National Disability-Heath survey and were anonymized prior to access.

### The Modified Rankin Scale (mRS)

**Figure 1:**
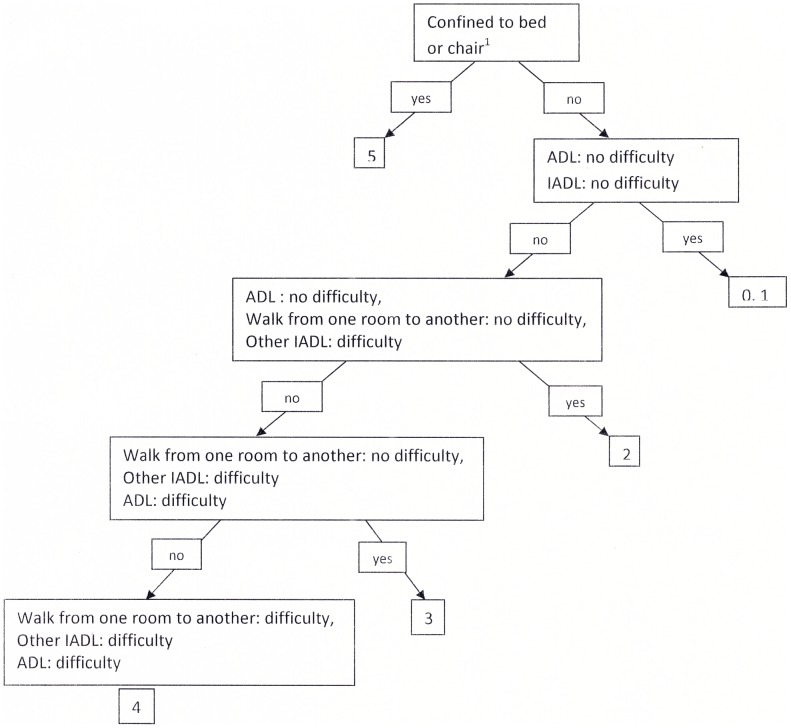
Scoring on the modified Rankin scale. ADL =  activities of daily living (washing, dressing, feeding, using the toilet); IADL =  instrumental activities of daily living (administrative procedures, managing medication, preparing a meal). ^1^- Subjects who could not transfer independently in or out of a bed or a chair and who could not walk independently indoors.

The Rankin scale is a “clinician reported measure” which evaluates global functional capacity following stroke. This scale is the most widely used in randomized clinical stroke trials. It is poorly sensitive to change but its psychometric properties are relatively good, particularly inter-rater reliability. The scale was not rated during the inquiry but was calculated for each subject from the results of the questions regarding independence in ADL (bathing, dressing, feeding, continence) and IADL (difficulty preparing a meal, carrying out administrative processes independently, managing medication and walking from one room to another) (Fig. 1)

### Weightings and analysis

First, a database including the variables of interest was constructed for each of the surveys. Subjects under 18 years of age were excluded. The databases (DHH and DHI) were then merged in order to represent the global French adult population living both at home and in institutions. In order to ensure a national representation, weightings were calculated by the Insee for both the DHH and the DHI surveys in order to take into account any unequal sample probabilities as well as non-responders. Adjustment variables included age, gender, marital status, health status measured during the DLH survey, type of accommodation and type of urban unit. All statistical analyses were carried out using statistical procedures adapted for complex survey plans (proc surveyfreq and proc surveylogistic, SAS Enterprise Guide version 4.3.), with stratifications and weightings taken into account. The prevalence of adults who reported a history of stroke and the prevalence of the different types of limitations compared to subjects who did not report a history of stroke were calculated with 95% confidence intervals. Chi squared tests were used for the bivariate analyses with the level of significance set at 0.05.

## Results

### Results of the DHH-DHI surveys (Table 1)

**Table 1:** Prevalence of self-reported history of stroke in the French adult population.

		Males		Females		p§	Together	
Prevalence whole population		Weighted prevalence	CI	Weighted prevalence	CI		Weighted prevalence	CI
All ages	1725	1.7	1.4–1.9	1.5	1.3–1.7	ns	1.6	1.4–1.7
≥50 years	1557	3.6	3.1–4.1	2.9	2.5–3.3	*	3.2	2.9–3.5
18–59 years	392	0.5	0.4–0.6	0.4	0.3–0.5	ns	0.4	0.4–0.5
60–74 years	422	3.4	2.5–4.3	2.4	1.7–3.1	ns	2.9	2.3–3.4
75–84 years	518	8.2	6.3–10.0	4.7	3.6–5.9	**	6.1	5.1–7.1
≥85 years	393	10.9	6.6–15.2	8.9	7.1–10.8	ns	9.5	7.7–11.4

§ : relationship between history of stroke and gender.

ns: not significant; *: p<0.05; **: p<10^−2^ ***: p<10^−3^; CI = Confidence interval.

The number of responders was 25 036 for DHH and 8 860 for DHI, making a total of 33 896 (15 092 males and 18 804 females). 73.0% of participants responded themselves, 10.9% with the help of a relative and 16.1% via a caregiver.

### Demographic results (Tables 1,2)

**Table 2:** Percentage of institutionalized adults with stroke, and time since stroke as a function of gender and age.

		Males		Females		p§	Together	
Percentage of institutionalized adults with stroke		%	CI	%	CI		%	CI
All ages	678	6.2	4.9–7.5	13.1	11.4–14.8	***	9.6	8.9–10.3
18–59 years	108	2.2	1.1–3.3	1.7	1.0–2.5	ns	2.0	1.4–2.6
60–74 years	98	4.5	2.6–6.3	2.9	1.3–4.4	ns	3.7	2.6–4.9
75–84 years	209	6.9	4.4–9.3	15.3	11.0–19.6	**	10.8	8.6–13.0
≥85 years	263	17.3	8.0–26.6	32.4	25.5–39.4	*	27.3	21.6-33.0
Time since stroke								
<2 years	341	28.6	21.6–35.7	18.6	14.2–23.0		23.7	19.3–28.2
2–4 years	411	22.8	17.7–27.9	27.4	21.5–33.3		25.0	21.2–28.9
5–9 years	395	19.2	14.0–24.5	27.9	21.7–34.1		23.5	19.4–27.5
≥10 years	456	29.3	23.3–35.3	26.1	19.5–32.8		27.8	23.3–32.2

The results of the DHH and DHI surveys showed a prevalence of self-reported stroke of 1.6% (CI95% [1.4%–1.7%]) in the global French adult population and of 3.2% in those aged 50 or more. Among those who reported a history of stroke, about 10% lived in institutions. The raw prevalence, not adjusted for age, was globally comparable in both genders (1.7% in males and 1.5% in females). The prevalence increased with age from 0.4% between 18 and 59 years to 9.5% in those aged 85 or more. The stroke had occurred more than 2 years prior to the survey for 75% of responders.

### Limitations in global activities (Table 3)

**Table 3:** Prevalence of self-reported ADl or iADL as a function of age and stroke history in France.

	18–59 years			60–74 years			75–84 years			85 years and over			All ages ≥18ans		
	S+	S-	RR	S+	S-	RR	S+	S-	RR	S+	S-	RR	S+	S-	RR
	%	%		%	%		%	%		%	%		%	%	
Overall limitation															
Not limited	1.5	82.3	0.2	31.9	63.0	0.5	13.1	39.3	0.3	12.5	18.2	0.6	19.5	74.1	0.3
Slightly limited	38.6	11.8	3.3	21.6	24.5	0.9	23.7	32.6	0.7	20.1	32.1	0.6	25.4	16.1	1.6
Severely limited	45.9	5.9	7.8	46.4	12.5	3.7	63.2	28.1	2.2	67.4	49.7	1.3	55.0	9.8	5.6
Difficulty to															
Wash independently	22.9	1.0	22.9	17.8	2.8	6.3	35.4	11.5	3.1	54.7	36.4	1.5	30.6	3.0	10.2
Dress and undress independently	18.7	1.1	17	15.1	2.7	5.6	33.4	10.0	3.3	49.6	27.3	1.8	27.3	2.7	10.1
Eat and drink independently	5.0	0.2	25	3.1	0.3	10.3	10.1	1.9	5.3	19.4	9.0	2.2	8.4	0.6	14
Use the toilet independently	7.0	0.4	17.5	7.7	0.7	11	17.8	3.6	4.9	33.8	15.8	1.1	15.1	1.1	13.7
Prepare meals independently	23.9	1.0	23.9	20.7	2.7	7.7	37.3	10.9	3.4	47.6	32.5	1.4	31.0	2.9	10.7
Carry out administrative processes independently	28.3	2.5	11.3	26.7	5.6	4.7	46.9	20.5	2.3	69.8	52.8	1.3	40.6	5.7	7.1
Take medication independently	9.9	0.6	16.5	13.5	1.2	11.3	30.5	6.7	4.6	44.4	24.4	1.8	23.3	1.8	12.9
Walk from one room to another independently	6.8	0.3	22.7	8.3	0.8	10.4	17.6	4.4	4	33.6	18.0	1.9	15.2	1.2	12.7

S+ = self-reported stroke, S- = no self-reported stroke, RR =  relative risk.

In response to the question: “do you feel that you have been limited for at least 6 months as a result of a health problem in activities which people normally do?”, 80.5% of participants with self-reported stroke replied yes and 55% said they were severely limited (compared with 25.9% and 9.8% in participants who did not report a history of stroke). The proportion of participants with self-reported stroke who felt limited, severely or not, was high in all the age groups analyzed but in comparison with participants who did not report a history of stroke, the difference was greater in those below the age of 60 (84.5% compared with 17.7%).

### Impact on ADL (Table 3)

Difficulties in carrying out ADL were more frequently reported in participants with self-reported stroke (between 8.4% and 30.6% depending on the ADL). The frequency increased with age in the participants with self-reported stroke, from 5% to 22.9% in those under the age of 60 compared with 19.4% to 54.7% for participants aged over 85 years. When all age groups were considered together, of the four ADL evaluated (washing, dressing, feeding and continence), washing was the most frequent problem reported by participants with self-reported stroke (30.6%)

### Impact on IADL (Table 3)

Difficulties in carrying out IADL were more frequently reported in participants with self-reported stroke (between 15.2% and 40.6% depending on the IADL). Between 6.8% and 28.3% of participants under the age of 60 with self-reported stroke reported difficulties in carrying out at least one IADL. This percentage increased from 33.6% to 69.8% for subjects over the age of 85 years. When all age groups were analyzed together, of the 4 IADL analyzed (difficulty preparing a meal, carrying out administrative processes independently, managing medication and walking from one room to another), difficulties in carrying out administrative processes independently was the most frequently reported (40.6% of participants with self-reported stroke).

### Relative risk of dependence

Before the age of 60, subjects who did not report a history of stroke rarely reported difficulties relating to independence (between 0.2 and 1.1% depending on the ADL and between 0.3 and 2.5% depending on the IADL) in contrast with participants of the same age with self-reported stroke. Even if the frequency of difficulties in carrying out ADL and IADL increased with age in the stroke population, the relative risk was comparatively much higher in younger participants than in older participants (17 to 25 times depending on the ADL for participants below the age of 60 and between 1.1 and 2.2 times for participants aged 85 and over).

### Modified Rankin Score (Table 4)

**Table 4:** Modified Rankin Scale scores as a function of stroke history, age and type of accommodation.

Estimated Rankin score	18–59		60–74		75–84		85 years and over		Total	
	S+	S-	S+	S-	S+	S-	S+	S-	S+	S-
	%									
Whole population										
0–1	60.3	96.6	67.8	92.0	42.0	73.4	21.3	38.7	50.4	92.6
2	15.3	2.0	11.7	4.1	16.9	12.2	19.0	19.5	15.3	3.6
3	17.6	1.1	12.3	3.1	23.4	10.1	25.9	23.9	19.1	2.7
4–5	6.8	0.3	8.3	0.8	17.7	4.4	33.8	18.0	15.3	1.2
Participants living at home										
0–1	61.3	96.9	70.3	92.6	46.5	75.8	28.0	46.4	55.3	93.5
2	15.1	1.9	11.6	3.9	18.1	12.1	23.8	21.5	16.0	3.4
3	17.5	1	11.7	2.9	23.5	9.4	29.3	23.5	19.0	2.4
4–5	6.0	0.2	6.4	0.6	12.0	2.7	18.9	8.6	9.7	0.7
Participants living in institutions										
0–1	10.0	19.2	3.1	13.4	5.1	9.2	3.4	6.8	4.2	10.8
2	24.3	32.7	12.1	23.2	7.2	13.6	6.1	10.8	8.0	17.6
3	22.2	19.6	28.8	29.5	22.6	26.0	16.8	25.7	20.5	24.9
4–5	43.5	28.4	56.0	33.9	65.1	51.3	73.7	56.8	67.3	46.7

S+ = self-reported stroke, S- = no self-reported stroke.

The frequency of participants with a mRS of 0 or 1 was quite high in the stroke population (50.4%). When all age groups were considered together, 34.4% of all participants with a history of stroke had a mRS of 3 or above (Moderate disability; requiring some help, but able to walk without assistance), compared with 3.9% of participants with no history of stroke. The proportion of severely disabled adults (mRS of 4 or above) was 15.3% in participants with self-reported stroke (compared with 1.2% of participants with no history of stroke).

In the participants with self-reported stroke who were under the age of 60 and living at home, 24.4% had a mRS of 3 or above (compared with 1.4% for those with no history of stroke). For those living in institutions, these frequencies were respectively 65.7% and 48.0%. When institutionalized participants and those living at home were analyzed together, 6.8% of participants below the age of 60 with self-reported stroke were severely disabled (mRS 4–5) compared with 0.3% in the rest of the studied population.

In the institutionalized participants, the level of dependence was severe and increased with age. Almost 75% of participants aged 85 years or more with self-reported stroke had a mRS of 4 or 5.

### Weight of stroke on dependence in the general population (data not presented)

**Figure 2:**
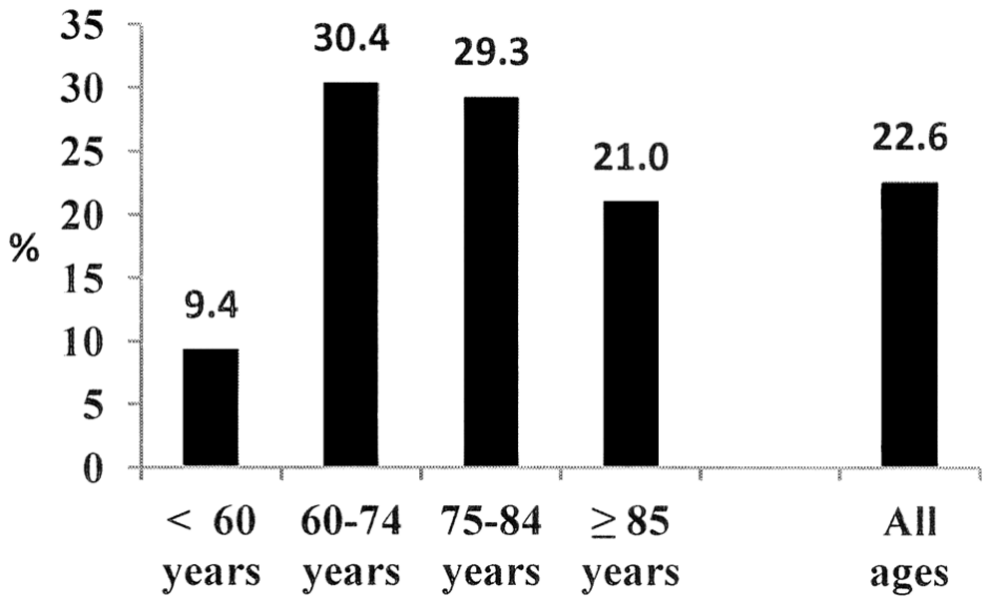
Prevalence of stroke history in subjects with a Rankin score of 5, as a function of age.

Overall, among all the participants who reported difficulties in an ADL, more than 1 in 10 reported a history of stroke. 14% of those who had difficulty dressing, 13.8% of those who had difficulty washing, 18.4% of those who had difficulty using the toilet and 18.8% of those who had difficulty eating and drinking reported a history of stroke. The proportions were similar for IADL: 10.2% had difficulty with administrative processes, 14.6% had difficulty with meal preparation, 16.9% had difficulty walking from one room to another and 17.3% had difficulty managing medication. The proportion was greater if there was a severe loss of independence. Thus, in the global heavily dependent population (classed as mRS 5), 22.6% reported a history of stroke. This proportion reached 30.4% for participants aged between 60 and 74 years of age and was 29.3% for those aged between 75 and 84 years (Fig. 2).

## Discussion

This survey is particularly novel because it established the proportion of stroke survivors in the French adult population and particularly, the level of dependence of stroke survivors was compared to that of persons with no history of stroke.

### Prevalence

The results showed a prevalence of stroke of 1.6% [1.4–1.7] in the French adult population. The prevalence of stroke in males in the DHH and DHI surveys was almost identical to that found in French males aged 50 or over in the SHARE survey (respectively 3.6 and 3.7%) but was lower for females in the DHH and DHI surveys (2.9% compared to 3.5% in French females aged 50 or over in the SHARE survey) [14]. In industrialized countries, the prevalence of stroke is generally a little higher [7]–[9], [15]. For example, in the United States, the prevalence of people with self-reported stroke (with or without sequelae) was 2.6% in the adult population in 2010 and had been relatively stable since 2006 [16]. This difference could be related to a lower incidence of stroke in France, particularly since the present study took institutionalized persons into account (almost 10% of the study population), which is rarely the case in cross sectional epidemiological studies [8].

### Disability

The analysis of disability took into account all stroke patients identified in the survey, however, the time since stroke differed between participants. The stroke had occurred less than two years previously in 20% and more than ten years previously in 25% of participants. The progressive occurrence of age-related comorbid factors, which could also cause disability, and the variability of changes over time could affect the analysis of specific stroke-related sequelae. Nevertheless, although it is difficult to compare data for prevalence and incidence, the levels of function found in the present study are similar to those described in two cohort studies: 26 to 31% of patients had an mRS between 3 and 5 [11], [12], which was the case for 34.4% in the present study.

The results of the present study therefore confirm, on a national level, that one in three stroke survivors are dependent.

### Younger subjects

Although the incidence of stroke in the younger population is lower than in the older population, it is not negligible (3 to 23 per 100 000 participants aged between 20 and 54 years) [17]. Moreover, Kissela et al. showed an increase in the proportion of 20–54 year olds with stroke in the USA (from 12.9% of the total number of first ever strokes in 1993–1994 to 18.6% in 2005) and a decrease in the average age of occurrence of first ever stroke (from 71.2 years to 69.2 years) [17]. In the present study, the prevalence in 18–59 year olds was 0.4% (which represents almost 140 000 people out of over 34 million aged between 20 and 60 years living in France in 2008).

In the literature, age appears to independently influence functional capacity but not neurological status, suggesting a greater capacity for compensation in younger subjects [18]. Neau et al. reported that 66 to 87% of young subjects with ischemic stroke become independent (mRS between 0 and 1 or 0 and 2) but only 56.7% have a quality of life evaluated as good [19]–[21]. In the present study, we found that 60.3% of participants under the age of 60 who reported having had a stroke had a mRS between 0 and 1. However, only 15.5% of those who reported a history of stroke declared having no limitation of function for less than 6 months because of a health problem in activities which people normally do (82.3% of those who did not report having stroke).

This difference is likely to be due to the perceptions of younger patients who have greater expectations in terms of the capacity to carry out activities. It could, however, also be due to the low sensitivity of the mRS for the evaluation of cognitive disability.

### Older subjects

In a large study, it was reported that only 19–24% of people over the age of 80 with self-reported stroke reach satisfactory functional levels (mRS 0–2) and that the short-term risk of mortality is high [22].

In the present study a very high prevalence of stroke was found in subjects aged 85 and over (9.5%). Subjects in this age group with self-reported stroke were frequently dependent (mRS 4–5: 33.8%). This was particularly the case for institutionalized participants, of which almost 75% of those with self-reported stroke were heavily dependent (only 3.4% had a mRS of 0 or 1).

### Institution versus home

The majority of participants with self-reported stroke lived at home (over 90% of cases). Among these, almost 10% were very heavily dependent (mRS 4 or5). Home help, such as early supported discharge which is necessary to compensate for a lack of independence, is currently insufficiently developed in France [23]–[25].

Older people with stroke are more likely to be institutionalized than younger people [18], [26], [27]. Black-Schaffer et al. reported that only 23% of stroke survivors over the age of 85 with a Functional Independence Measure (FIM) score below 40 returned home compared with 60% of subjects with a similar FIM score but aged under 55 years [28].

However, there are currently no specific studies on institutionalized patients. Moreover, this population is only rarely taken into account in cross sectional epidemiological surveys (surveys are generally carried out in people's homes).

The results of the present study found that almost 1 in 10 people with self-reported stroke lived in institutions. All ages considered together, these people were highly dependent; 87.8% had a mRS of 3 or above (compared with 28.7% of those with self-reported stroke living at home) and two thirds were confined to bed or chair (67.3%). The proportion was 43.5% for 18–60 year olds and reached 73.7% in those aged 85 or older.

### History of stroke versus no stroke

The results clearly showed that the relative risk of loss of independence, depending on the presence or not of self-reported stroke, was greater for younger subjects for both ADL and IADL. Although the proportion of participants who reported difficulties in ADL and IADL increased with age in the whole of the population studied, the relative risk of functional limitations decreased. As a result, the risk was 11 to 25 times higher in those under the age of 60 with self-reported stroke than in participants of the same age with no history of stroke. However, the risk was only 1.5 to 2.1 times more frequent in those aged over 85 years (and was between 2 and 5 times more frequent for the 75–84 year olds). This puts the literature which reports a poor functional prognosis for elderly people following stroke into perspective, since their potential for independence is quite similar to people with no history of stroke in the same age group. However the good functional prognosis often described for younger stroke survivors needs to be re-considered. 84.5% of those under the age of 60 who reported a history of stroke also reported that they were “limited because of a health problem in activities which people normally do” (despite the fact that they had a mRS of 0 or 1 in 60.3% of cases). This limitation is probably the result of neuropsychological impairments related to the stroke which are often under evaluated in the younger population.

### Limitations of the study

The study was based on a declarative survey using face to face interviews (and not telephone interviews). As a result, the diagnosis of stroke in the studied population cannot be certain.

There may have been an overestimation of the prevalence of stroke in the younger population who may have confused stroke with a TIA, cerebral palsy or a severe traumatic brain injury. Conversely, in the elderly population or in persons with severely disabling cognitive or communication disorders, the prevalence may have been underestimated (through difficulty in obtaining the medical history or difficulty in filling in the initial questionnaire etc.). Lastly, participants with a history of stroke but few sequelae (isolated attentional or behavioural problems, for example), may not have declared a history of stroke and are therefore probably under represented within the sample. However, in terms of prevalence the results are similar to other surveys such as the SHARE study and the recent work by Feigi et al. (which estimated the prevalence of stroke as 6.4% in persons over the age of 75, compared with 7% in our study). The use of a questionnaire to estimate the prevalence of stroke is frequent in epidemiological studies, with a relatively robust level of sensitivity and specificity [29].

It is important to consider the fact that, it is not because a subject reported a history of stroke that any disability reported is the direct result of the stroke. Moreover, patients with a history of stroke often have many other potentially disabling pathologies (diabetes, heart attack, etc. However, it is also not possible to be certain that in other cohort studies stroke was the only cause of dependence, particularly for older subjects.

Equally, because this was a declarative survey, it was not possible to identify the different impairments of the persons interviewed (particularly cognitive, communication or behavioural disorders) in a reliable manner. For this reason, the study focused on the description of restrictions in ADL and IADL in those who reported a history of stroke.

With regard to the impact of stroke in terms of dependence, this was also based on declarative data. Moreover, in 22.3% of cases, a caregiver responded on behalf of the subject. However, because of the large size of the population studied and the very dependent nature of the subjects who were unable to self-report, this should have had little impact on the quality of the responses to the questions on ADL and IADL.

Lastly, the mRS was not carried out directly. It was estimated from the responses given in the questionnaire. A simplified version of the Rankin scale has recently been developed (Simplified Modified Rankin Scale Questionnaire -SmRSq) [30] which allows the mRS to be estimated from 5 closed questions. The questions asked are very similar to the questions asked in this study (for example: “can you walk from one room to another without help from another person?”). The calculation of the mRS via such closed questions, similar to the questions we asked, has been considered to be reliable [31]. However, the mRS has never been validated in the general population.

## Conclusion

This study reports the level of independence of French adults who reported a history of stroke on a national level. The results showed that there is a high prevalence of stroke in older subjects and that this pathology has important functional consequences, particularly in people under the age of 60. The incidence of stroke is increasing in younger people and the French population is aging. It is therefore necessary to improve follow-up care in order to improve independence and social participation in younger people and to facilitate older, often heavily dependent people, to stay at home. The National action plan STROKE 2010–2014 should help to improve coordination between general practitioners, neurologists, physiatrists and the paramedical staff who manage these patients.
